# Frequency-Dependent Relationship Between Resting-State fMRI and Glucose Metabolism in the Elderly

**DOI:** 10.3389/fneur.2019.00566

**Published:** 2019-05-29

**Authors:** Fangyang Jiao, Zhongzhan Gao, Kuangyu Shi, Xize Jia, Ping Wu, Chengfeng Jiang, Jingjie Ge, Hui Su, Yihui Guan, Shenxun Shi, Yu-Feng Zang, Chuantao Zuo

**Affiliations:** ^1^Department of Nuclear Medicine, Daping Hospital, Army Medical University, Chongqing, China; ^2^Center for Cognition and Brain Disorders, Institute of Psychological Sciences, Hangzhou Normal University, Hangzhou, China; ^3^Zhejiang Key Laboratory for Research in Assessment of Cognitive Impairments, Hangzhou Normal University, Hangzhou, China; ^4^Department of Nuclear Medicine, Klinikum Rechts der Isar, Technische Universität München, Munich, Germany; ^5^PET Center, Huashan Hospital, Fudan University, Shanghai, China; ^6^Shanghai Mental Health Center, Shanghai Jiaotong University, Shanghai, China; ^7^Psychiatry Department, Huashan Hospital, Fudan University, Shanghai, China; ^8^Institute of Functional and Molecular Medical Imaging, Fudan University, Shanghai, China; ^9^Human Phenome Institute, Fudan University, Shanghai, China

**Keywords:** fMRI, PET, FDG, frequency-dependent, multi-modalities

## Abstract

Both glucose metabolism and resting-state fMRI (RS-fMRI) signal reflect hemodynamic features. The objective of this study was to investigate their relationship in the resting-state in healthy elderly participants (*n* = 18). For RS-fMRI signal, regional homogeneity (ReHo), amplitude of low frequency fluctuations (ALFF), fractional ALFF (fALFF), and degree of centrality (DC) maps were generated in multiple frequency bands. Glucose uptake was acquired with ^18^F-fluorodeoxyglucose positron emission tomography (FDG-PET). Linear correlation of each pair of the FDG-PET and RS-fMRI metrics was explored both in across-voxel way and in across-subject way. We found a significant across-voxel correlation between the FDG-PET and BOLD-fMRI metrics. However, only a small portion of voxels showed significant across-subject correlation between FDG-PET and BOLD-fMRI metrics. All these results were similar across all frequency bands of RS-fMRI data. The current findings indicate that FDG-PET and RS-fMRI metrics share similar spatial pattern (significant across-voxel correlation) but have different underlying physiological importance (non-significant across-subject correlation). Specifically, FDG-PET measures the mean glucose metabolism over tens of minutes, while RS-fMRI measures the dynamic characteristics. The combination of FDG-PET and RS-fMRI provides complementary information to reveal the underlying mechanisms of the brain activity and may enable more comprehensive interpretation of clinical PET-fMRI studies. Future studies would attempt to reduce the artifacts of RS-fMRI and to analyze the dynamic feature of PET signal.

## Introduction

Blood-oxygenation-level dependent (BOLD) resting-state functional magnetic resonance imaging (RS-fMRI) and positron emission tomography (PET) are two functional neuroimaging techniques that have been demonstrated to hold significant value for diagnosis of human function and have elucidated neurobiological processes (1–3). The RS-fMRI signal depends on the cerebral blood volume, cerebral blood flow (CBF), and the cerebral metabolic rate of oxygen (CMRO^2^) (4, 5). It's a relative and indirect measure of brain activity. FDG-PET is a standard method for measuring human brain glucose metabolism (6, 7). These BOLD and FDG-PET measurements may together provide insight into brain activity patterns. Several studies have investigated the relationship between glucose metabolism and BOLD signal activity using combined PET/fMRI imaging techniques (8–10). For example, Aiello and colleagues investigated the correlation between regional glucose metabolism and regional RS-fMRI metrics in two ways, i.e., subject-wise across-voxel correlation and voxel-wise across-subject correlation (10). They found that the subject-wise across-voxel correlation showed significant similar spatial pattern between glucose metabolism and RS-fMRI metrics, however, only a few brain regions showed significant voxel-wise across-subject correlation (10).

RS-fMRI has relatively high temporal resolution (usually 2 s) and therefore it supports frequency-dependent analysis. However, to the best of our knowledge, all existing correlation studies of PET and RS-fMRI have utilized only a single conventional frequency band 0.01–0.08 Hz. However, some studies suggested the need to consider more specific frequency bands in the BOLD-fMRI studies of brain activity (11, 12). The current study aimed to investigate the relationship between relative glucose uptake (rGU) assessed by FDG-PET and four metrics derived from resting-state BOLD-fMRI, i.e., amplitude of low frequency fluctuations (ALFF), fractional amplitude of low frequency fluctuations (fALFF), regional homogeneity (ReHo), and degree of centrality (DC), of a few sub-frequency bands.

## Materials and Methods

### Participants

A total of 20 healthy elderly right-handed participants were recruited from the nearby community of Huashan Hospital, Fudan University. Each subject underwent a single MRI first and then PET scan within 1 day. Subjects were not included from the study if they had medication use that could interfere with brain function, had contraindications to MRI, or history of neurological or psychiatric illness. In addition, subjects were not included in the study if any abnormality was present on the PET and/or MRI scan. The protocol was approved by the Research Committee at the Huashan Hospital, Fudan University. All subjects gave written informed consent in accordance with the Declaration of Helsinki. Two subjects were excluded for further analysis due to large head motion during BOLD-fMRI scanning (see data processing), and hence, 18 elderly subjects (13 women and 5 men; average age, 61.5 ± 6.4 years; all were right-handed) were included finally.

### Image Acquisition

MRI scans were performed using a 3-T General Electric Signa scanner (GE, USA). Resting-state fMRI was obtained with the following parameters: TR = 2,000 ms, TE = 35 ms, flip angle = 90°, slice number = 45, field of view (FOV) = 240 × 240 mm, voxel size = 3.3 × 3.3 × 4.0 mm^3^, 240 time points (8 min). During the scanning, subjects were instructed to keep still with their eyes closed but not to fall asleep. High-resolution 3D T1-weighted anatomical images in the sagittal orientation were acquired with a magnetization-prepared rapid gradient-echo sequence (repetition time = 2,300 ms, echo time = 2.98 ms, flip angle = 9°, FOV = 256 × 256 mm^2^, matrix size = 256 × 256, slice thickness = 1 mm, without interslice gap, voxel size = 1 × 1 × 1 mm^3^ and 176 slices).

PET scans were performed with a Siemens Biograph 64 PET/CT (Siemens, Germany) in 3D mode. First, these subjects were still and resting in a room that was quiet and had dim lighting. Then an intravenous bolus injection of 185 MBq of FDG was administered. CT transmission scan was performed to allow attenuation correction. Next, The PET scan was started 45 min after the injection and lasted 10 min. Hanning filters were used with filtered-back projection for image reconstruction, resulting in a transaxial and axial cut-off frequency of 0.5. Additional technical details on the scanner were reported elsewhere (13, 14).

### Data Processing

The FDG-PET data was performed by statistical parametric mapping 8 package (SPM8, the Wellcome Department of Neurology, London U.K.) software implemented in Matlab8.0 (Mathworks Inc, Sherborn, MA). Scans from each subject were spatially normalized into MNI brain space with linear and nonlinear 3D transformations. The transformation from single subject space to MNI was derived from 3D-T1 image by new segmentation (Ashburner, 2007) implemented in SPM8. The normalized PET images were then smoothed with a Gaussian filter of 6 mm FWHM over a 3D space to increase the signal to noise ratio to facilitate statistical analysis. To keep the standardization procedure the same as RS-fMRI metrics (see below), the glucose uptake of each individual voxel was divided by the global mean uptake, and then the relative glucose uptake (rGU) was obtained (15).

In addition, all PET data were analyzed with MR-based partial volume correction (PVC) using the PMOD software tool (version 3.7, PMOD Technologies Ltd., Zürich, Switzerland, Muller-Gartner method) and then processed again as above.

The BOLD-fMRI data were processed using SPM8 and Data Processing Assistant for Resting-State fMRI (DPARSF) toolkits (16). The first 10 time points were removed to avoid non-equilibrium effects of magnetization and to allow the subjects to adjust to the scanning noise. Subsequent images were corrected for slice-timing and realigned within the session. Subjects with an estimated maximum head motion larger than 3.0 mm or 3.0° were excluded from additional analysis (two subjects were excluded). The T1-weighted images were then co-registered to the mean BOLD-fMRI image using a process of rigid-body transformation. This was followed by a process of spatial normalization using a nonlinear transformation and the SPM8 T1 template to convert the image to Montreal Neurological Institute (MNI) space. The BOLD-fMRI images were spatially normalized too by employing the same normalization parameters as used for the T1 image and re-sampled to 3 × 3 × 3 mm resolution. Spatially smoothing was performed with a 6 × 6 × 6 mm full width at half maximum (FWHM) Gaussian kernel. It should be noted that the spatial smoothing procedure was different for ALFF calculation and the other two metrics (ReHo and DC). For ALFF and fALFF analyses, the data were spatially smoothed before ALFF or fALFF calculation. But for ReHo and DC, the spatial smoothing was performed immediately after ReHo and DC calculation (described below). To minimize very low-frequency drifts, linear trending was removed. The Friston 24-parameter movement correction method was applied using multiple linear regression analysis. The Friston-24 was used to regress out head motion effects (17). This correction method is more effective than other methods such as correction for rigid-body using six parameters, derivative 12 parameters, or voxel-specific 12 regressors (18). Because this correction is not universally accepted, we analyzed the data both with and without removal of head motion parameters. Finally, the scrubbing procedure was performed (18–20).

The ALFF was calculated as described previously (21). The time courses for each individual voxel were subject to a fast Fourier transformation to the frequency domain and the power spectrum was determined. The square root of this spectrum was calculated for each frequency and then averaged across 0.01–0.08 Hz. This averaged square root was used as an ALFF index. In addition to the conventional low-frequency band of 0.01–0.08 Hz, we also calculated ALFF in 4 sub-bands as previously defined (3), i.e., slow-5 (0.01–0.027 Hz), slow-4 (0.027–0.073 Hz), slow-3 (0.073–0.198 Hz), and slow-2 (0.198–0.25 Hz). For standardization purpose, the ALFF value of each voxel was divided by the global mean ALFF.

The fALFF was calculated as the ratio of the amplitude within the low-frequency range (0.01–0.08 Hz) to the total amplitude over the full frequency range (0–0.25 Hz in our study). The fALFF indicates the relative contribution of oscillations in the low frequency range to the signal variations over the whole frequency range (22). In addition to the conventional frequency band (0.01–0.08 Hz), the fALFF of 4 sub-bands were calculated fALFF as in ALFF analysis. The standardization procedure was the same as the above.

ReHo measures the local synchronization or similarity of the time courses of nearest neighboring voxels (usually 27 voxels) (23). After preprocessing, BOLD fMRI data was temporally filtered into a conventional low frequency band (0.01–0.08 Hz) and 4 sub-bands as above. The ReHo was calculated using Kendall's coefficient of concordance (KCC) of the time series of every 27 neighboring voxels, and then the KCC value (ranged from 0 to 1) was given to each center voxel. Next, the ReHo map was spatially smoothed with a 6 × 6 × 6 mm FWHM Gaussian kernel. As above, the ReHo value of each voxel was divided by the global mean ReHo for standardization.

DC is a metric that assesses the summed functional connectivity of each individual voxel with all voxels of the brain (24). After preprocessing of the image, band-pass filtering (a conventional low frequency band and 4 sub-bands as above). All pair-wise Pearson correlation coefficients were calculated and the cutoff for correlation coefficient was set to 0.25 (25). The weighted DC map was obtained and then was spatially smoothed with a 6 × 6 × 6 mm FWHM Gaussian kernel. The DC value of each voxel was divided by the global mean DC for standardization.

### Statistical Analysis

For each metric (rGU, ALFF, fALFF, ReHo, and DC), we performed one-sided one-sample *t*-tests against a fixed value of 1 (the global mean value after standardization) to identify the brain regions showing significantly higher value than the global mean (15). Voxels with a *P-*value < 0.001 and cluster size >351 mm^3^ (corrected with AlphaSim multiple comparison correction, corresponding to a corrected significance level of *P* < 0.05).

The across-voxel correlation analysis was performed for each pair of metrics of each subject by Pearson's correlation. It should be noted that the *n* for across-voxel correlation analysis here was the total number of voxels within the entire brain (*n* = 70,831 voxels). One-sample *t*-test was performed on the 18 correlation coefficients to explore whether the mean correlation coefficient was significantly different from zero using SPSS (SPSS, version 19.0, Inc., Chicago). For multiple comparison correction, a stringent threshold of *P* < 0.001 was utilized.

The across-subject correlation analysis was performed for each pair of metrics of each voxel. It should be noted that the *n* for across-subject correlation analysis here was the total number of participants (*n* = 18). The multiple comparison correction was performed using Monte Carlo simulations (individual voxel *P* < 0.05 and volume > 6,156 mm^3^, corresponding to a corrected significance level of *P* < 0.05). Given the fact that 3 previous PET-fMRI correlation studies (10, 26, 27) did not find consistently significant across-subject correlation between PET and RS-fMRI metrics, we did not used very stringent correction in order to find more consistent across-subject correlation.

## Results

**Figure 1:**
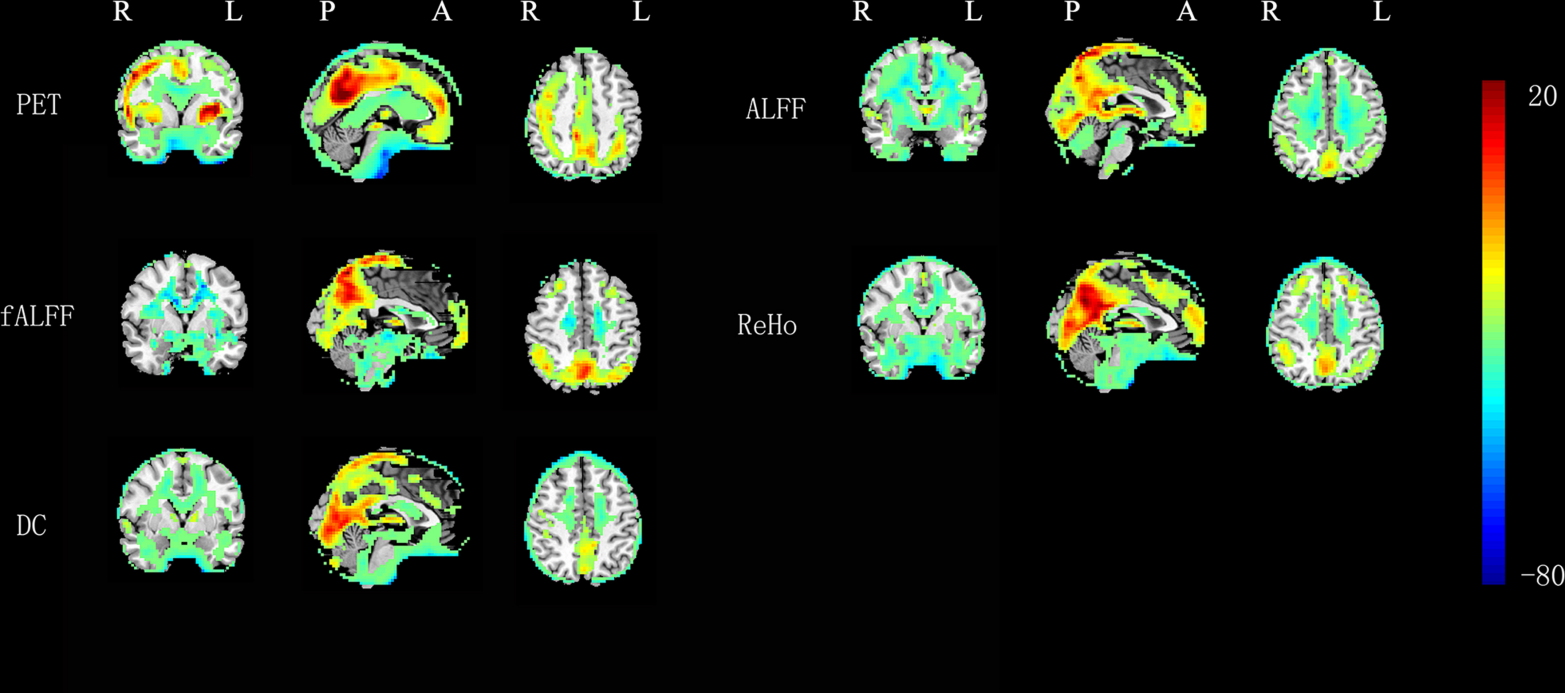
One-sample *t*-tests on RS-fMRI and FDG-PET metrics in 0.01–0.08 Hz (*P* < 0.05, AlphaSim corrected at cluster level).

The overall spatial patterns of one-sided one-sample *t*-tests were consistent for all the 5 metrics (rGU, ALFF, fALFF, ReHo, and DC), i.e., significantly higher brain activity in the default mode network regions than the global mean value (Figure 1). Those regions included the posterior cingulate cortex, medial prefrontal cortex, inferior parietal lobule, and precuneus, primarily within the default mode network. However, significantly higher values can also be seen in superior temporal gyrus, middle occipital gyrus, lingual gyrus, and so on.

### Across-Voxel Correlation

The across-voxel correlations between all metrics at individual level are shown in Table 1. For the RS-fMRI conventional frequency band of 0.01–0.08 Hz, the correlation coefficients between ReHo and rGU were the best (mean *r* = 0.527). The correlation between the FDG-PET and BOLD-fMRI metrics is mild but significant (*P* < 0.001). The BOLD-fMRI measurements showed significantly higher correlations with each other (ALFF vs. fALFF, ALFF vs. ReHo, ALFF vs. DC, fALFF vs. ReHo, fALFF vs. DC, and ReHo vs. DC) than with rGU (i.e., rGU vs. ALFF, vs. fALFF, vs. ReHo, and vs. DC).

**Table 1:** The mean value and standard deviation of across-voxel correlation coefficients without removing head motion parameters in 18 subjects (*P < 0.001).

**Band**	**0.01–0.08**	**0.01–0.027**	**0.027–0.073**	**0.073–0.198**	**0.198–0.25**
PET/ALFF	0.198 ± 0.123*	0.207 ± 0.120	0.190 ± 0.128*	0.053 ± 0.058	−0.028 ± 0.082
PET/fALFF	0.306 ± 0.136	0.253 ± 0.136	0.261 ± 0.128	−0.150 ± 0.126*	−0.278 ± 0.135*
PET/ReHo	0.527 ± 0.152*	0.488 ± 0.157*	0.513 ± 0.163*	0.487 ± 0.146*	0.293 ± 0.151*
PET/DC	0.429 ± 0.147*	0.359 ± 0.127*	0.357 ± 0.125*	0.356 ± 0.156*	0.283 ± 0.165*
ALFF/fALFF	0.576 ± 0.101*	0.673 ± 0.063*	0.541 ± 0.103*	0.177 ± 0.282	0.330 ± 0.234*
ALFF/ReHo	0.559 ± 0.133*	0.585 ± 0.088*	0.554 ± 0.154*	0.389 ± 0.109*	0.369 ± 0.102*
ALFF/DC	0.413 ± 0.157*	0.366 ± 0.113*	0.378 ± 0.148*	0.405 ± 0.143*	0.319 ± 0.157
fALFF/ReHo	0.674 ± 0.099*	0.649 ± 0.076*	0.673 ± 0.108*	−0.051 ± 0.183*	0.172 ± 0.138*
fALFF/DC	0.548 ± 0.113*	0.427 ± 0.135*	0.509 ± 0.112*	0.648 ± 0.148*	0.089 ± 0.197*
ReHo/DC	0.721 ± 0.067*	0.624 ± 0.083*	0.689 ± 0.071*	0.648 ± 0.147*	0.458 ± 0.199*

For the RS-fMRI metrics in sub-bands, the correlation coefficients in the low-frequency band were larger than those in the high-frequency band (Table 1). The correlation coefficients between ReHo and rGU were the best (mean *r* = 0.513) in 0.027–0.073 Hz. Interestingly, the ALFF and fALFF values of the high-frequency band negatively correlated with some other metrics. The fALFF contrast is significantly correlated with rGU, showing the best negative correlation coefficients (mean *r* = −0.278) at 0.198–0.25 Hz.

### Across-Subject Correlation

**Figure 2:**
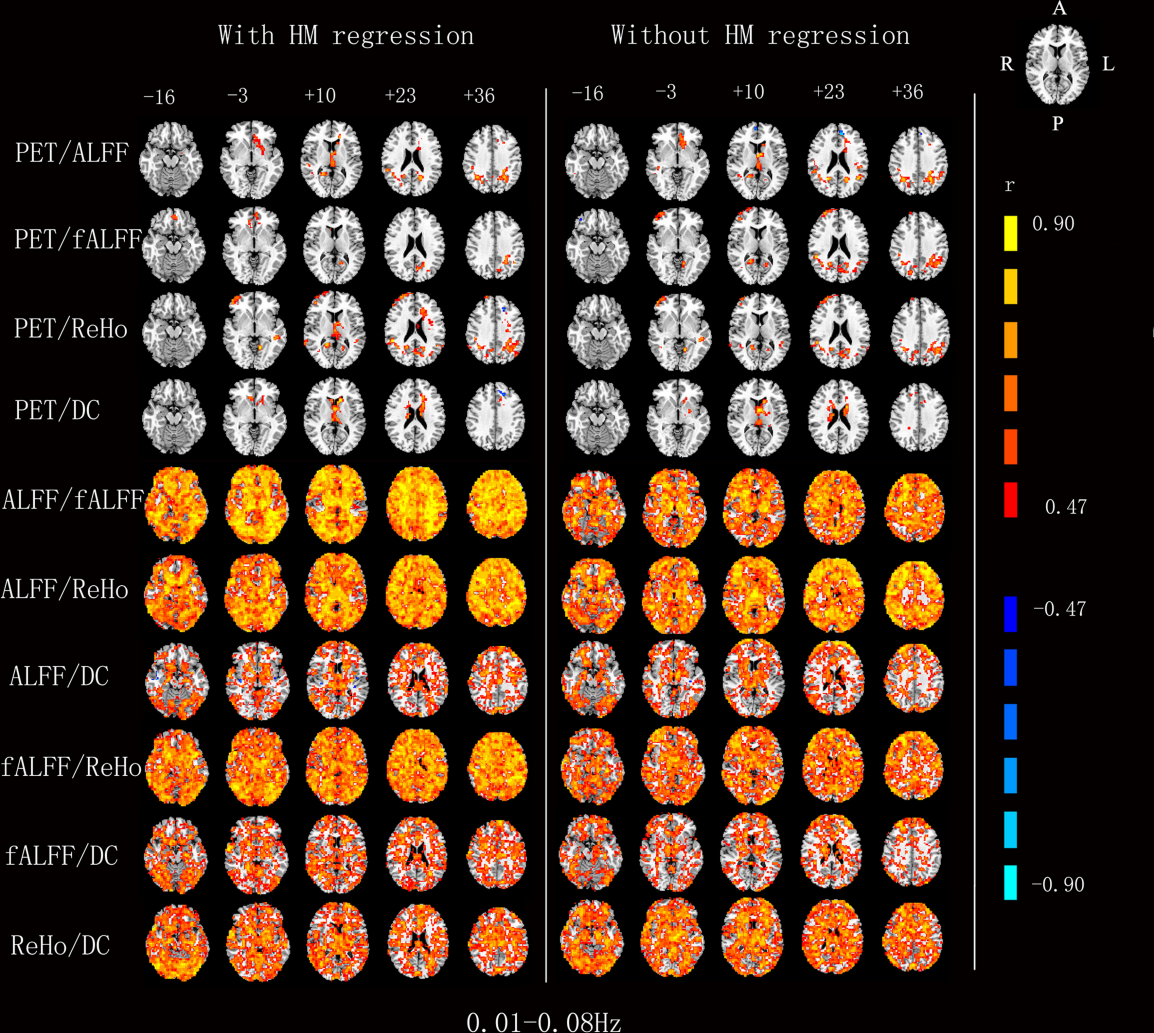
Voxels showing a significant correlation (*P* < 0.05, AlphaSim corrected at cluster level) between the different metrics in 0.01–0.08 Hz. The color scale represents *r* values ranging from 0 to 1.

In general, only a few brain regions showed significant correlation (corrected *P* < 0.05) across subjects between rGU with each of the 4 RS-fMRI metrics (Figure 2). And to further look at these regions, most voxels located in the white matter and some voxels were near the ventricles. However, the 4 RS-fMRI derived metrics, i.e., ALFF, fALFF, ReHo, and DC, showed significant correlations (corrected *P* < 0.05) with each other in most brain regions.

**Figure 3:**
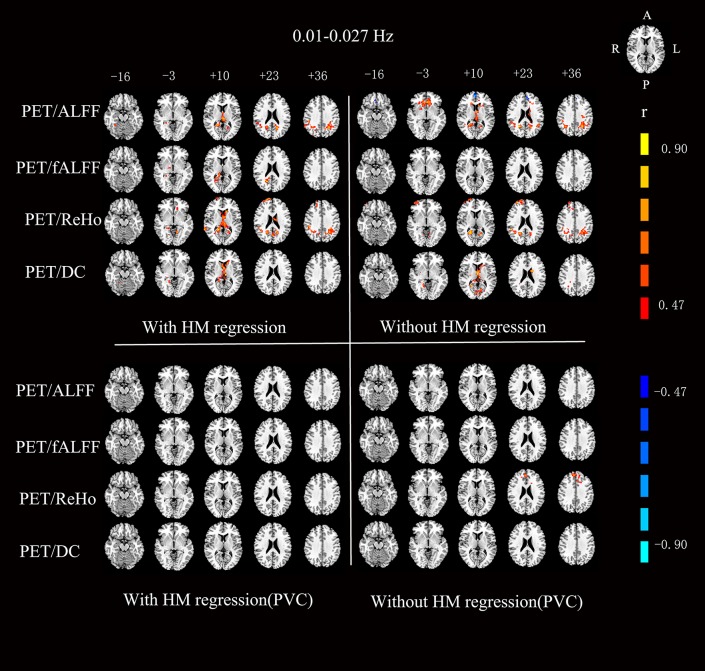
Voxels showing a significant correlation (*P* < 0.05, AlphaSim corrected at cluster level) between the different metrics in 0.01–0.027 Hz. The color scale represents *r* values ranging from 0 to 1. HM, head motion; PVC, partial volume correction.

**Figure 4:**
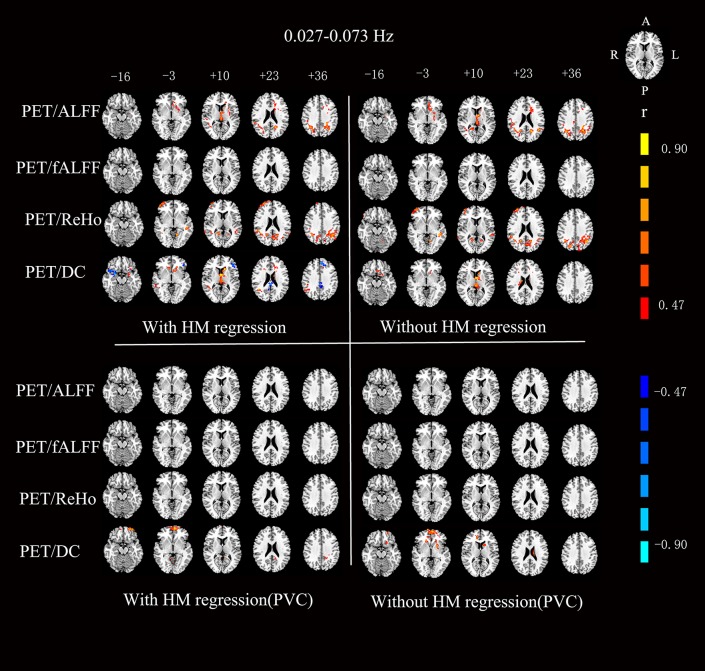
Voxels showing a significant correlation (*P* < 0.05, AlphaSim corrected at cluster level) between the different metrics in 0.027–0.073 Hz. The color scale represents *r* values ranging from 0 to 1. HM, head motion; PVC, partial volume correction.

**Figure 5:**
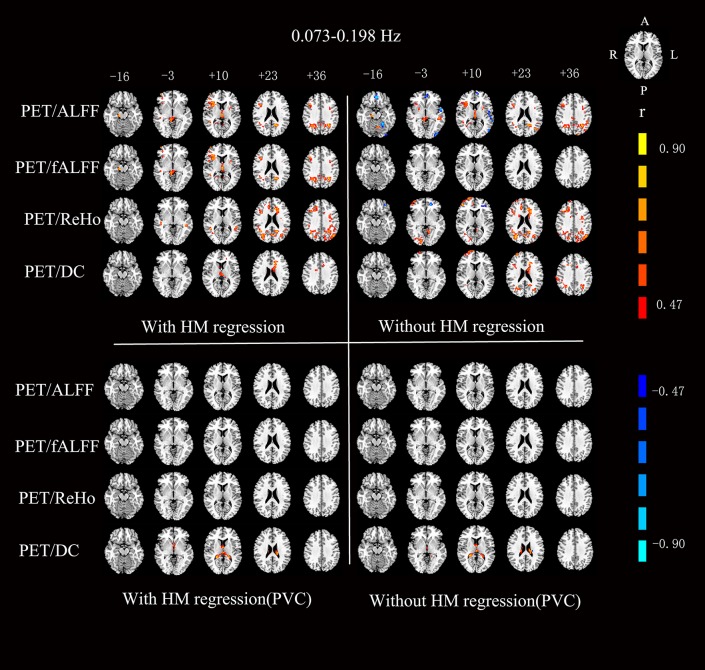
Voxels showing a significant correlation (*P* < 0.05, AlphaSim corrected at cluster level) between the different metrics in 0.073–0.198 Hz. The color scale represents *r* values ranging from 0 to 1. HM, head motion; PVC, partial volume correction.

**Figure 6:**
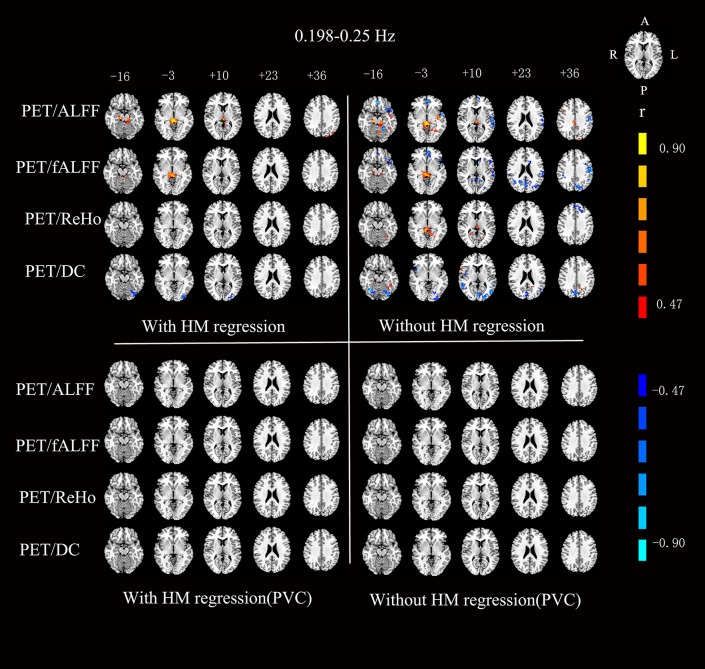
Voxels showing a significant correlation (*P* < 0.05, AlphaSim corrected at cluster level) between the different metrics in 0.198–0.25 Hz. The color scale represents *r* values ranging from 0 to 1. HM, head motion; PVC, partial volume correction.

For the RS-fMRI metrics in sub-bands, the across-subject correlation analysis also showed that only a small portion of voxels showed significant correlation between FDG-PET and BOLD-fMRI modalities (Figures 3–6). Even some RS-fMRI metrics did not show any correlation with PET.

### Head Motion Analysis

After head motion regression, the mean across-voxel correlation coefficient of the 18 subjects was in general higher than without head motion regression (Table 2). However, the results of across-subject correlation were similar for with vs. without head motion correction by visual inspection (Figures 3–6).

**Table 2:** The mean value and standard deviation of across-voxel correlation coefficients with removing head motion parameters in 18 subjects (*P < 0.001).

**Band**	**0.01–0.08**	**0.01–0.027**	**0.027–0.073**	**0.073–0.198**	**0.198–0.25**
PET/ALFF	0.198 ± 0.123	0.207 ± 0.120	0.190 ± 0.128	0.053 ± 0.058	−0.028 ± 0.082
PET/fALFF	0.306 ± 0.136*	0.253 ± 0.136	0.261 ± 0.128*	−0.150 ± 0.126*	−0.278 ± 0.135*
PET/ReHo	0.527 ± 0.152*	0.488 ± 0.157	0.513 ± 0.163*	0.487 ± 0.146*	0.293 ± 0.151*
PET/DC	0.429 ± 0.147*	0.359 ± 0.127*	0.357 ± 0.125*	0.356 ± 0.156*	0.283 ± 0.165*
ALFF/fALFF	0.576 ± 0.101*	0.673 ± 0.063*	0.541 ± 0.103*	0.177 ± 0.282*	0.330 ± 0.234
ALFF/ReHo	0.559 ± 0.133*	0.585 ± 0.088*	0.554 ± 0.154*	0.389 ± 0.109*	0.369 ± 0.102*
ALFF/DC	0.413 ± 0.157*	0.366 ± 0.113*	0.378 ± 0.148*	0.405 ± 0.143*	0.319 ± 0.157*
fALFF/ReHo	0.674 ± 0.099*	0.649 ± 0.076*	0.673 ± 0.108*	−0.051 ± 0.183	0.172 ± 0.138*
fALFF/DC	0.548 ± 0.113*	0.427 ± 0.135*	0.509 ± 0.112*	0.648 ± 0.148*	0.089 ± 0.197
ReHo/DC	0.721 ± 0.067*	0.624 ± 0.083*	0.689 ± 0.071*	0.648 ± 0.147*	0.458 ± 0.199*

### Partial Volume Correction (PVC) for PET

**Figure 7:**
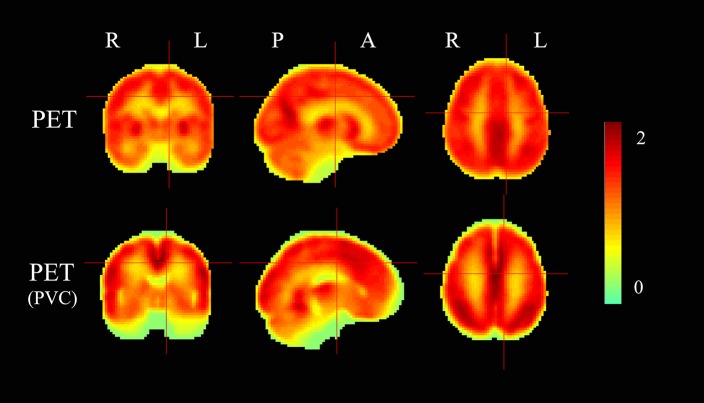
The mean PET maps before and after partial volume correction (PVC). The color scale represents relative glucose uptake values.

The mean PET data before and after PVC were shown in Figure 7. The across-voxel correlation coefficients after PVC for PET were slightly smaller than without PVC (Tables 3, 4). The across-subject correlation analysis also showed decreased correlation after PVC for PET (Figures 3–6).

**Table 3:** The mean value and standard deviation of across-voxel correlation coefficients without removing head motion parameters in 18 subjects after PVC for PET (*P < 0.001).

**Band**	**0.01–0.08**	**0.01–0.027**	**0.027–0.073**	**0.073–0.198**	**0.198–0.25**
PET/ALFF	0.047 ± 0.080*	0.044 ± 0.072*	0.048 ± 0.084	−0.053 ± 0.055	−0.113 ± 0.076
PET/fALFF	0.211 ± 0.077*	0.121 ± 0.083*	0.208 ± 0.082*	−0.045 ± 0.102	−0.187 ± 0.077*
PET/ReHo	0.421 ± 0.061*	0.381 ± 0.056*	0.411 ± 0.067*	0.378 ± 0.055*	0.279 ± 0.104*
PET/DC	0.388 ± 0.082*	0.328 ± 0.062*	0.341 ± 0.074*	0.236 ± 0.129*	0.189 ± 0.168

**Table 4:** The mean value and standard deviation of across-voxel correlation coefficients with removing head motion parameters in 18 subjects after PVC for PET (^*^P < 0.001).

**Band**	**0.01–0.08**	**0.01–0.027**	**0.027–0.073**	**0.073–0.198**	**0.198–0.25**
PET/ALFF	0.031 ± 0.077	0.025 ± 0.067	0.035 ± 0.083	−0.043 ± 0.056	−0.093 ± 0.067
PET/fALFF	0.174 ± 0.097*	0.089 ± 0.075*	0.185 ± 0.110*	−0.061 ± 0.099	−0.182 ± 0.070*
PET/ReHo	0.395 ± 0.070*	0.352 ± 0.059*	0.391 ± 0.073*	0.381 ± 0.069*	0.327 ± 0.098*
PET/DC	0.343 ± 0.091*	0.298 ± 0.072*	0.322 ± 0.080*	0.223 ± 0.147*	0.222 ± 0.133*

## Discussion

This work was designed to provide a comprehensive overview of the correlation of metabolism as measured with FDG-PET and changes in BOLD-fMRI metrics. It is the first study, to our knowledge, to systematically evaluate the correlation of fMRI measures of different frequency bands and glucose metabolism.

### Across-Voxel Correlation and Across-Subject Correlation

Our findings of the overall significant across-voxel correlation mean that the spatial pattern of regional glucose metabolism was similar to that of RS-fMRI metrics. Although the current RS-fMRI and PET were not acquired simultaneously (the tests were conducted a few hours apart in the same day), our findings are consistent with previous results (8, 10, 26). Specifically, the regional glucose metabolism and RS-fMRI metrics showed higher value in the brain regions in the default mode network.

In contrast to the overall significant correlation of across-voxel correlation, i.e., similar spatial distribution between FDG-PET and RS-fMRI maps, the across-subject analysis found that only a small percentage of voxels showed significant correlation between PET and fMRI maps. The low across-subject correlation was consistent with two previous studies which also utilized regional RS-fMRI metrics (10, 27). In addition, the brain regions showing significant across-subject correlation between FDG-PET and RS-fMRI regional metrics were not well-overlapped for the 3 studies (the two previous studies, and the current study) by visual inspection.

The above seemingly paradoxical correlations (across-voxel vs. across-subject) have a few possible interpretations as following.

The first possible explanation for the lack of correlation of the two sets of imaging results is non-linear coupling between glucose and oxygen metabolism. Such non-linear coupling was previously observed for CBF, oxygen consumption, and ATP production in the task state (28). Both PET and fMRI methods can be sensitive to different components of metabolic-hemodynamic coupling. ^18^F-FDG PET is an indicator of glucose metabolism, and RS-fMRI imaging results from complex relationships of hemodynamic parameters, including CBF, CBV, and CMRO2.

The second explanation is the dynamic characteristics of RS-fMRI. FDG-PET measures an integrated or mean glucose metabolism over time, while the RS-fMRI metrics reflect the dynamic characteristics of BOLD signal over time. As the unit of BOLD signal is relative, the mean RS-fMRI signal over time is not physiologically meaningful. Arterial spin labeling (ASL) is an fMRI technique to non-invasively measure cerebral blood flow (CBF). Some pulsed ASL sequences generate a CBF time course. In such case, both the mean CBF and CBF dynamic metrics (e.g., ReHo or ALFF) characteristics can be calculated. An early study has shown that the mean CBF, CBF-ReHo, and CBF-ALFF were significantly higher in the brain regions of default mode network than other regions (29). Another ASL study found that the mean CBF did not show significant across-subject correlation with BOLD ReHo nor BOLD ALFF (30). These evidences suggest that the mean value of glucose metabolism or CBF over time are quite different from the dynamic characteristics (e.g., ReHo and ALFF) over time. Our studies measured the mean glucose metabolism at the voxel level over time. In contrast, RS-fMRI measures the dynamic fluctuations of brain activity at the voxel level, reflecting the temporal characteristics of spontaneous activity.

### Frequency-Dependent Analysis

Previous simultaneous PET-fMRI recording studies have focused on only a single frequency band of 0.01–0.08 Hz. Since the first RS-fMRI study on sub-frequency bands by Zuo et al. (3), many studies have revealed frequency-dependent alterations in brain disorders (31–33). The current study found frequency-dependent across-voxel correlation between glucose metabolism and RS-fMRI metrics. Specifically, the two low frequency bands (0.01–0.027 Hz, 0.027–0.073 Hz) showed more significant across-voxel correlation, while the correlations were less significant and even not significant for the higher frequency bands of RS-fMRI (Table 1). For the across-subject correlation analysis, few brain regions showed significant correlation for all frequency bands. Although it has been accepted that the higher frequency RS-fMRI signal is more contributed by physiological noise (e.g., heart beat and respiration), many studies have shown the physiological (34) and pathophysiological importance of higher frequency RS-fMRI signal (35, 36). PET FDG represents an integration of glucose metabolism over time (e.g., 10 min), while the RS-fMRI signal reflects frequency-dependent fluctuation of brain activity over time. In fact, the temporal resolution of PET image acquisition could be as high as fMRI (e.g., every 2 s). However, frequency dependent PET analyses for resting state FDG-PET have not yet been reported and could be an important topic for future PET studies.

### The Effects of Head Motion Regression and Partial Volume Correction

We analyzed the correlation between glucose metabolism and RS-fMRI metrics with and without performing head motion regression. We found that the influence of head motion showed no prominent contribution to either the across-voxel correlation or across-subject correlation.

The potential impact of partial volume effect (PVE) for the quantification of PET imaging has been acknowledged (37). PVE is a term used to describe two phenomena that degrade the quantitative accuracy of PET data (38). A given voxel may contain multiple tissue types, with the resulting voxel value representing the average signal from these fractional contributions. The mean signal does not necessarily represent the true distribution of the radiotracer accurately. To investigate the PVE on the results, we corrected PVE on PET data. The results showed that PVE generally had very slight effects on the correlation results. It should be noted that the PVE is also a matter for RS-fMRI data, especially for the ReHo and DC because the ReHo or DC value of any voxel is largely overlapped with that of its neighbors due to the computational nature of ReHo and DC. However, there has been no widely accepted PVC method for fMRI. It should be investigated in future studies.

### Limitations

There are several limitations of this study. First, for standardization purposes, the current study used the whole brain mean glucose metabolism to divide that of each voxel. This is also the way of standardization procedure in many RS-fMRI studies (15, 23). But clinical PET investigation usually takes a region of interest (ROI), where the glucose metabolism is considered stable, e.g., cerebellum or brain stem, as reference. Future studies would test to what extent the standardization processes will affect the results. Second, the current study enrolled a relatively small number of subjects. This issue is more concerned for across-subject correlation analysis. Third, the subjects were all elderly and therefore our findings may be not generalized to other populations. Forth, PET and MRI images were not acquired simultaneously. Fifth, this exploratory study uses a linear correlation index, which cannot fully reflect the complex and potentially non-linear interactions of glucose metabolism with BOLD dynamic characteristics. Sixth, the less consistent across-subject correlation might be partly due to the physiological noise of RS-fMRI. Future studies should attempt more procedures to correct the physiological noise of RS-fMRI.

## Conclusion

Being consistent with two previous studies, we found significant across-voxel correlation, while less significant across-subject correlation between regional glucose metabolism and regional RS-fMRI metrics. In addition, we found that the higher frequency band of RS-fMRI showed less across-voxel correlation with FDG-PET than lower frequency bands. PET and RS-fMRI could provide a more comprehensive understanding of the involved mechanisms and facilitate diagnosis and treatment.

## Ethics Statement

This study was carried out in accordance with the recommendations of the Research Committee at the Huashan Hospital, Fudan University with written informed consent from all subjects. All subjects gave written informed consent in accordance with the Declaration of Helsinki. The protocol was approved by the Research Committee at the Huashan Hospital.

## Author Contributions

CZ and Y-FZ designed experiments. FJ, CJ, JG, and PW carried out experiments. FJ analyzed experimental results. ZG and XJ analyzed data and developed analysis tools. KS, HS, YG, and SS assisted with experiments. FJ wrote the manuscript.

### Conflict of Interest Statement

The authors declare that the research was conducted in the absence of any commercial or financial relationships that could be construed as a potential conflict of interest.
